# Omega-3 Fatty Acids and Muscle Strength—Current State of Knowledge and Future Perspectives

**DOI:** 10.3390/nu16234075

**Published:** 2024-11-27

**Authors:** Maja Tomczyk

**Affiliations:** Department of Biochemistry, Gdansk University of Physical Education and Sport, 80-336 Gdansk, Poland; maja.tomczyk@awf.gda.pl

**Keywords:** omega-3 fatty acids, muscle strength, sarcopenia, eicosapentaenoic acid, docosahexaenoic acid

## Abstract

Omega-3 polyunsaturated fatty acids (*n*-3 PUFAs), specifically the most biologically active (eicosapentaenoic acid (EPA) and docosahexaenoic acid (DHA)), have well-documented health-promoting effects, including, but not limited to, controlling inflammatory processes in the human body and supporting cardiovascular and cognitive health and visual processes. There is also some evidence pointing to the potential of EPA and DHA to preserve and/or enhance muscle strength. Muscle strength plays a crucial role in preventing age-related decline in skeletal muscle mass and function and the subsequent impaired functionality in the daily lives of the elderly. It also has a significant role in physical performance by aiding in the effectiveness of training elements, such as vertical jumps, sprinting, changes of direction, rate of force development, and anaerobic conditioning. Previous studies have indicated that supplementation with *n*-3 PUFAs may have a small but significant effect on preserving and/or increasing muscle strength in both healthy adults and in elderly. However, the number of studies published on this topic is limited. The goal of this narrative review is to summarize the effects of EPA and DHA supplementation on muscle strength and identify the limitations of previous studies that, if addressed, will help confirm or exclude the beneficial effects of *n*-3 PUFAs on muscle strength in humans.

## 1. Introduction

**Figure 1:**
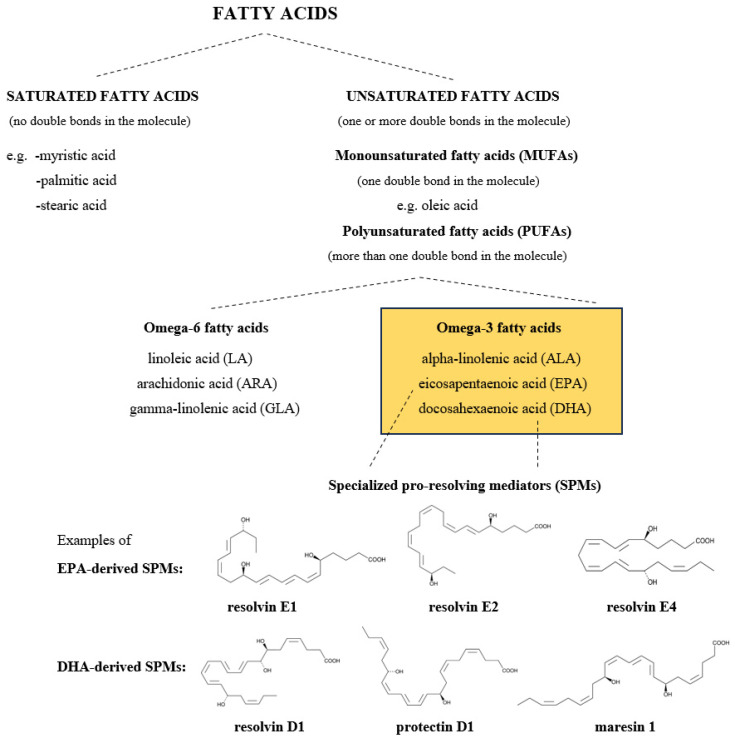
General classification of fatty acid families, including *n*-3 PUFAs and their derivatives.

The term *n*-3 PUFAs includes three main fatty acids, alpha-linolenic acid (ALA), eicosapentaenoic acid (EPA), and docosahexaenoic acid (DHA), belonging to the PUFA family and carrying the first double bond on the third carbon atom from the methyl end of the fatty acyl chain (Figure 1). Due to the fact that the human body has no omega-3 desaturase-specific enzyme responsible for inserting a double bond in the “*n*-3” position of the fatty acyl chain, *n*-3 PUFAs cannot be synthesized de novo in humans and have to be obtained from the diet or by supplementation [1,2]. ALA, a precursor of the *n*-3 PUFAs family, can be found in plant foods such as green leaves, flaxseeds, some nuts (e.g., walnuts), and some vegetable oils (e.g., flaxseed oil, canola oil, and soybean oil). In contrast to ALA, the best source of EPA and DHA is fatty fish, particularly salmon, mackerel, trout, and canned sardines [3,4]. The EPA and DHA contents of some selected foods are shown in Table 1.

Although ALA can undergo metabolic conversion to longer-chain EPA and DHA, such conversion is substantially limited [5]. For example, in a study by Pawlosky et al. [5], where participants of both sexes were administered a strict diet with a controlled fatty acid composition, followed by an isotope tracer of α-linolenate, it was estimated that the conversion of ALA to EPA and DHA was 0.2% and 0.05%, respectively.

In 2004, Harris and von Schacky [6] proposed the omega-3 index (O3I), the sum of EPA and DHA as a percentage of total fatty acids in erythrocytes, as a marker of both EPA and DHA intake and coronary heart disease risk. Since then, O3I has been used to evaluate the status of *n*-3 PUFAs in people globally. It was estimated that the majority of countries of the world, including many countries in North America and Europe, Turkey, South Africa, China, and Australia, have low O3I (4–6%). In countries such as Spain, France, Denmark and Sweden, Tunisia, Mongolia, Taiwan, and New Zealand, a moderate O3I (6–8%) has been observed. The desirable O3I (>8%) has been demonstrated in the fewest number of regions, including Iceland, Norway, Finland, South Korea, Japan, Alaska, and Greenland [7,8,9,10,11]. There are a number of studies indicating that an increased intake of EPA and DHA, and consequently improved O3I, has beneficial effects on cardiovascular and cognitive health and visual processes [12,13,14,15,16,17].

The favorable effects of EPA and DHA are strongly related to their anti-inflammatory actions. Through interacting with lipid rafts in cell membranes, they inhibit the activation of NFκB, a key transcription factor that upregulates genes coding for inflammatory proteins. EPA and DHA have been shown to reduce the expression of, for example, tumor necrosis factor (TNF)-alpha, interleukin (IL)-6, IL-8, IL-1β, and cyclooxygenase-2 (COX-2) [18]. Moreover, they are also substrates from which specialized pro-resolving mediators (SPMs) are formed. These bioactive lipid mediators, which include resolvins, protectins, and maresins (Figure 1), have a crucial role in the resolution of inflammation [19,20].

The beneficial health-promoting effects of *n*-3 PUFAs may also include, as shown in several studies, maintaining or increasing muscle strength in individuals of various age groups and physical activity [21,22,23,24,25].

The purpose of this narrative review is to summarize selected reports on the effects of EPA and DHA supplementation on muscle strength. In addition, it aims to identify the limitations of previous studies that, if addressed, will help confirm/exclude the beneficial effects of *n*-3 PUFAs on muscle strength in humans.

## 2. *n*-3 PUFAs and Muscle Strength in Adults

Muscle strength plays an important role in both overall health in general population and sports performance in athletes [26]. In a meta-analysis involving 1,907,580 participants, a higher muscle strength was associated with a lower mortality risk in an adult, apparently healthy population, regardless of age and follow-up period [27]. It was also inversely correlated with the frequency of cardiovascular disease, type 2 diabetes, metabolic syndrome, and cognitive functioning [28,29,30,31]. Moreover, grip strength has been identified as a biomarker of aging and mortality [32].

Muscle strength is also a critical motor ability that underpins the effectiveness of training elements, such as vertical jumps, sprints, changes in direction, rate of force development, and anaerobic conditioning. Moreover, a greater muscular strength was associated with decreased injury rates [26]. There are a limited number of studies evaluating the effects of *n*-3 PUFA supplementation on muscle strength parameters in young adults [33], which seems to be a promising direction for further research.

In the longest experiment to date, Heileson et al. administered 2.275 g of EPA + 1.575 g of DHA daily or safflower oil as a placebo at 4.5 g a day during a 10-week strength training program conducted three times a week by amateur athletes of both sexes [21]. At the beginning and at the end of the experiment, the researchers assessed body composition using electrical bioimpedance and evaluated muscle strength parameters: 1-repetition maximum barbell back squat (1RMSQT) and bench press (1RMBP). An increase in absolute and relative 1RMBP and relative 1RMSQT was observed in participants taking EPA and DHA. Simultaneously, an increase in O3I by 109.7% from a value of 4.5% in the *n*-3 PUFAs group was noted with no changes in the placebo group. However, no strength training-induced changes in lean body mass were observed, contrary to the authors’ hypothesis [21]. The most important trials in this field are presented in Table 2. Several studies address the benefits of *n*-3 PUFAs, but there are some inconsistencies in the findings.

### Neuromuscular Adaptations: Potential Mechanism of n-3 PUFA Action in Adults

The evaluation of LBM using densitometry and muscle strength (isokinetic/eccentric maximum voluntary contraction (MVC) of the knee flexors and unilateral 1-repetition maximum (1 RM) for leg extension) was also performed in the study by Philpott et al. [22]. In this 6-week experiment, resistance-trained men were subjected to a 2-week weight loss using a 40% energy deficit and simultaneous supplementation with 2 g of EPA and 2 g of DHA per day or placebo throughout the whole study. Participants taking EPA + DHA showed a significant decrease in body weight with no change in LBM and an increase in 1RM leg extension compared to baseline values [22].

The authors of both this and the previous study highlighted changes in neuromuscular function as one of the potential mechanisms that could explain their observations. This phenomenon was also investigated in two studies by Lewis et al. [34,35]. In the first study, young male athletes were supplemented with 375 mg of EPA, 230 mg of DPA, and 510 mg of DHA or placebo for 21 days. At the beginning of the experiment, after various performance tests and, analogously, after the supplementation period, quadricep MVCs were assessed with electromyography (EMG) recordings. Researchers showed an increase in the EMG of the vastus lateralis, the largest part of the quadriceps by 20 ± 18%, and an unclear effect on MVC force (4.1  ±  6.6%) in the participants taking *n*-3 PUFAs [34]. In a second experiment, the researchers evaluated neuromuscular adaptations to sprint interval training (SIT) in recreationally active males during a 2-week training period combined with *n*-3 PUFA supplementation (375 mg EPA, 230 mg DPA, 510 mg DHA, and 5000 IU retinyl palmitate and vitamin D3) or placebo [35]. In contrast to previous results and the authors’ hypothesis, there was no improvement in neuromuscular adaptations measured by changes in quadriceps MVC force in the group following supplementation with *n*-3 PUFAs. According to the authors, this may be caused by the duration of supplementation or intense training stimulus that suppressed the effect of supplementation [35].

An increase in 1 RM of knee extension with a concomitant increase in LBM was seen in a study by Crestani et al., where young male strength training amateurs were supplemented ~1.4 g of *n*-3 PUFAs per day or placebo. The beneficial effects of the 28-day supplementation were seen only in the group following the *n*-3 PUFA supplementation [36]. Contrary to this, no effects on maximal strength, speed, or power, despite improved anaerobic endurance capacity, were seen in soccer players during 4-week training combined with *n*-3 PUFAs at a dose of 0.1 g/kg of supplement [37].

Finally, in a recent systematic review and meta-analysis involving 14 studies, Santo Andre et al. concluded that *n*-3 PUFA supplementation may have a very small yet significant effect on muscle strength in healthy young and older adults with no impact on muscle mass and its function. However, the authors of the study emphasized the low certainty of the findings and suggested a strict methodology and data reporting as necessary components of future research to allow the confirmation of these results [25]. In addition to the studies included by the authors in the review, it is worth mentioning the recent publications by Heileson et al. [21] and Xu et al. [23]. Given the adequate length and dose of EPA and DHA, and the randomized, placebo-controlled designs, they provide important arguments to support the beneficial effects of these acids on muscle strength.

## 3. *n*-3 PUFAs and Muscle Strength in the Elderly

There are data indicating that *n*-3 PUFAs may also increase muscle mass and strength in the elderly, and consequently slow the development of sarcopenia [42,43]. Sarcopenia is defined as a progressive decline in skeletal muscle mass and function, associated with a higher risk of falls and bone fractures, increased disability, prolonged hospitalization, postoperative complications, metabolic disorders, as well as increased mortality [44]. Sarcopenia is estimated to affect about 10–16% of the elderly worldwide; however, its prevalence is higher in patients with comorbidities [45]. Hence, slowing its development through, for example, improved nutrition and/or supplementation is important for a better quality of life for the elderly.

In a randomized controlled trial (RCT) conducted by Xu et al., participants aged >60 years were administered 4 g/day of fish oil capsules for 6 months [23]. At the end of the study, subjects receiving *n*-3 PUFAs showed an increase in the total skeletal muscle mass and appendicular skeletal muscle mass, compared to the placebo group receiving 4 g/day of corn oil. Moreover, an increase in muscle strength was shown based on the hand grip strength test. In addition, the authors conducted a Timed Up and Go time test, which confirmed an improvement in physical performance. According to the authors of the experiment, this effect could be linked to an increase in the rate of muscle protein synthesis (MPS) [23].

Smith et al. indicated that this outcome may be related to the effect of *n*-3 PUFAs on the augmented expression of the mechanistic target of the rapamycin complex 1 (mTORC1) pathway [38]. However, scientific papers published in recent years, including a 2024 meta-analysis, seem to deny such a mechanism of action. Therdyothin et al. reported that the consumption of *n*-3 PUFAs can increase the rate of protein synthesis throughout the body, but such an effect was not strictly noted for rates of MPS. In view of this, it is believed that other mechanisms underlie the beneficial effects of *n*-3 PUFAs on increasing muscle mass and strength [46].

### 3.1. Neuromuscular Adaptations: Potential Mechanism of n-3 PUFA Action in the Elderly

Rodacki et al. showed that improvements in muscle strength in the elderly may be related to the effects of *n*-3 PUFAs on neuromuscular conduction [24]. In this study, 45 elderly women (aged 64 ± 1.4 years) were divided into three groups. The first group performed only strength training for 90 days, while the second group performed the same training combined with fish oil supplementation in a total amount of 2 g/day. The final group started supplementation 60 days before the start of strength training and continued it during the following 90 days combined with training, for a total of 150 days. An increase in the peak torque and rate of torque development for knee flexor and extensor, plantar, and dorsiflexor were visible in all studied groups. However, this effect was statistically significantly higher in the groups receiving fish oil [24].

**Figure 2:**
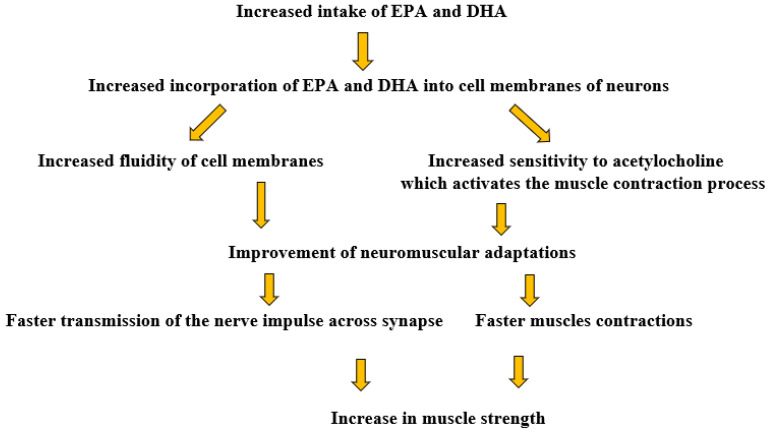
Potential mechanism of EPA- and DHA-induced increases in muscle strength based on Rodacki et al. [24].

On the basis of EMG, a greater muscle activation was noted in the fish oil-supplemented groups, confirming the contribution of *n*-3 PUFAs to increasing muscle strength through their effect on neuromuscular conduction. The potential mechanism of EPA- and DHA-induced increases in muscle strength based on Rodacki et al. [24] is presented in Figure 2. The authors of the experiment indicated that *n*-3 PUFAs may improve muscle function by altering the fluidity of the membrane and affecting sensitivity to acetylcholine, a neurotransmitter that is involved in triggering muscle contraction. As a result, *n*-3 PUFAs may mediate a faster synaptic transmission at the neuromuscular junction. The increase in membrane fluidity caused by the consumption of *n*-3 PUFAs and their incorporation into cell membranes may also contribute to the improved transmission of nerve impulses [24]. Moreover, *n*-3 PUFA-induced changes in lipid composition may affect the reuptake and release of neurotransmitters, which may also influence the effect seen. Some of these mechanisms have been demonstrated in animal models [47,48]; however, they need to be confirmed in human studies.

In the study by Rodacki et al., women supplemented with *n*-3 PUFAs also improved functional capacity, which was confirmed by the “chair-rising” test. However, these differences were not found between the period of *n*-3 PUFA supplementation nor in the results in the other tests: “foot up and go”, “sit and reach”, and “6-minute walk” between groups [24]. A recently published review article by Phillips et al. also indicates that the efficacy of *n*-3 PUFAs in improving muscle strength in the elderly population is probably related to neuromuscular functions [49].

### 3.2. Meta-Analyses on n-3 PUFAs and Muscle Strength in the Elderly and Some Important Points About Supplementation

Importantly, several meta-analyses published in recent years confirm the effectiveness of *n*-3 PUFAs in improving musculoskeletal health in the elderly. For example, Tseng et al. published a network meta-analysis of randomized controlled trials, which confirmed that *n*-3 PUFAs increased upper limb muscle strength and lower limb fitness; the highest efficacy was noted when *n*-3 PUFAs were used in amounts exceeding 2.5 g/day. The authors of this work also showed that *n*-3 PUFA supplementation had no significant effect on skeletal muscle mass [50].

Similarly, a meta-analysis published by Bird et al. 3 years earlier revealed that *n*-3 PUFA supplementation had a beneficial effect on MVCs, indicating an increase in muscle strength [42]. However, the authors pointed a paucity of studies conducted on patients with sarcopenia and a small number of participants in studies. Moreover, several studies were at risk of bias because of a lack of blinding or randomization, or had failures in reporting these factors, and the authors suggested larger studies with a rigorous control of the sources of bias to provide more conclusive results [42].

Researchers also showed increases in skeletal muscle mass, which stands in opposition to the results obtained by Tseng et al. The aforementioned differences may be due to the varying number of the studies included in the meta-analyses as well as the use of different methods of data analysis. In addition, the results of a meta-analysis by Cornish et al. revealed that *n*-3 PUFA supplementation resulted in a significant increase in lower body strength. However, such an effect was not observed for the upper body [51]. This is likely due to the fact that, as pointed by Candow and Chilibeck, muscles in the lower body are more susceptible to aging, compared to muscles in the upper body [52]. In a narrative review by Rossato et al., including young and older adults, the authors pointed to the equivocal effects of *n*-3 PUFA supplementation on muscle function in the elderly and no clear consensus regarding its effect in adults [53]. However, after 2018, by which the publications in the review were considered, the results of several important studies, including meta-analyses, suggested beneficial effects on *n*-3 PUFAs regarding muscle strength in these two age groups [21,22,23,25,46,49,50].

Among the mechanisms of action responsible for the beneficial effects of *n*-3 PUFAs on muscle strength, their anti-inflammatory properties should also be mentioned. A 2023 meta-analysis confirmed that the dietary inflammatory index (DII) was associated with sarcopenia [54]. According to Diao et al., the risk of sarcopenia significantly increased (OR = 1.22) for each 1-point increase in the DII score [54]. In contrast, Custodero et al. confirmed that *n*-3 PUFA supplementation alleviated chronic low-grade inflammation in middle-aged and older adults. The authors of the meta-analysis reported a statistically significant reduction in C-reactive protein (CRP) (−0.17 mg/L) [39]. It is also worth mentioning a study by Jang et al., who compared serum *n*-3 PUFAs in elderly subjects with diagnosed sarcopenia (71.9 ± 4.7 years old) and elderly subjects without sarcopenia (68.6 ± 6.5 years old). The researchers showed that those with sarcopenia had significantly lower *n*-3 PUFA concentrations. Moreover, linear regression analyses confirmed a positive association between serum *n*-3 PUFAs and grip strength (β = 13.130), providing an additional argument for a beneficial effect of *n*-3 PUFAs on muscle strength [41].

In the context of improving muscle function and mass in the elderly, the additional use of protein in conjunction with *n*-3 PUFAs may magnify the achieved results. For example, Haß et al. reported an increase in muscle power in community-dwelling older adults (65–85 years of age) who received a dietary intervention that included algae oil (2.19 g EPA + DHA), as well as a 300 mL whey drink (containing 27 g protein) [40]. The subjects in this study were instructed to achieve a protein intake of 1.2–1.5 g/kg bw/d and, at the same time, to include additional 25–30 g of protein in each meal. All participants in the experiment took vibration exercises on a vibration plate once a week, as well as being instructed to perform home-based resistance exercises [40].

The favorable effects of *n*-3 PUFA supplementation on muscle strength and mass are not supported by all research. For example, a study by Krzymińska-Siemaszko showed that the administration of *n*-3 PUFAs (1.3 g/d over 12 weeks) to the elderly people with decreased muscle mass did not result in an increase in muscle strength and mass, compared to the control group [55].

Although the vast majority of studies use EPA and DHA, there are some data regarding the importance of ALA in regulating muscle mass and strength in the elderly. In the 12-week study undertaken by Cornish and Chilibeck, ALA in the form of flaxseed oil (14 g/d) was administered to elderly individuals (65.4 ± 0.8 years) in conjunction with a resistance training program [56]. The results indicated that the addition of ALA to the training program had only a minor effect on muscle strength, muscle thickness, and lean tissue mass in men and women. Moreover, there was no reduction in the concentration of IL-6 in the women. This may be due to the reduced conversion of ALA to EPA and DHA as a result of decreased estrogen levels in the women studied, the high levels of which enhance the aforementioned conversions [56]. Hence, it seems that ALA is not an effective ingredient in the prevention and supportive treatment of sarcopenia. Nevertheless, further studies are needed in this aspect due to the scarce data to date.

### 3.3. Suggestions for n-3 PUFA Dosage in the Elderly

There are several suggestions in the literature regarding the dosage of *n*-3 PUFAs in the context of the prevention and treatment of sarcopenia. For example, based on a review of the scientific literature, Giacosa et al. recommended consuming ≥500 mg/day of EPA + DHA to prevent or help treat sarcopenia [57]. Hawley and Baum emphasized that, in order to maintain skeletal muscle mass in the elderly, it may be necessary to consume EPA + DHA above the Dietary Guidelines for Americans, which is currently 450–500 mg/day [58]. In a meta-analysis including 10 randomized, controlled trials with a total of 552 participants aged 60 years and older, an increase in muscle strength was observed only with a dose higher than 2 g/day [59]. The researchers observed that, at these doses, applied especially over 6 months, *n*-3 PUFAs can promote muscle mass and improve walking speed in participants [59]. It is worth noting that these doses are consistent with the results obtained in the above-mentioned meta-analyses, and recommendations for *n*-3 PUFA supplementation in the context of prevention and/or treatment of sarcopenia may be much higher than the recommendations dedicated to the general population.

## 4. Potential Risks of *n*-3 PUFA Supplementation

Despite a number of beneficial effects from *n*-3 PUFA supplementation, the potential negative consequences of their application should be mentioned. These include increased risk of the most common chronic cardiac arrhythmia, atrial fibrillation (AF), increased risk of bleeding, gastrointestinal disturbances, and concerns about lipid peroxidation. The meta-analyses by Albert et al. and Lombardi et al. with a total of 12 RCTs showed that supplementation with *n*-3 PUFAs was associated with an increased risk of AF, particularly when high doses of *n*-3 PUFAs were applied [60,61]. This contradicts a report by Qian et al. that examined 17 cohorts for a potential association of incident AF with EPA, DHA, and docosapentaenoic acid (DPA) concentrations in blood and adipose tissue. This study did not reveal an association between the concentrations of these acids and increased risk of AF [62]. Although the mechanism for the potential effect of *n*-3 PUFAs on the development of AF remains unclear [60] and current findings are inconsistent, the potential risk of developing AF, particularly in high-risk patients, should be considered before the decision to begin *n*-3 PUFA supplementation is made.

In contrast to AF, the evidence confirming the lack of effect of *n*-3 PUFAs on prolonging bleeding time is consistent [63,64,65]. For example, in an analysis by Jeanson et al. including eight different clinical trials of *n*-3 PUFA-enriched enteral nutrition products, no increased risk of bleeding was observed with either short-term doses of up to 10 g/day of EPA + DHA or long-term (up to 52 weeks) doses above 1.5 g/day [63]. Questions about lipid peroxidation arise from the specifics of the polyunsaturated bonds in EPA and DHA, which are easily oxidized, leading to the production of lipid peroxides that are detrimental to cell integrity. The addition of antioxidants and proper storage of supplements should protect against potential oxidation. Moreover, according to the European Food Safety Authority (EFSA) position, long-term EPA and DHA supplementation up to 5 g a day does not seem to increase the risk of spontaneous bleeding, bleeding complications, or lipid peroxidation [66].

Finally, one of the most frequently described consequences of *n*-3 PUFA supplements is a fishy aftertaste or fishy ‘burps’. A meta-analysis of 21 RCTs of prescription *n*-3 PUFAs products providing 1.8 to 4 g EPA + DHA/day for a period from 6 weeks to 5 years to a total of 24,460 participants, indicated that the use of *n*-3 PUFA products is generally safe and without serious adverse effects. However, an increase in fishy aftertaste, fishy ‘burps’, and nausea was observed with *n*-3 PUFAs compared to the placebo [67]. Although these are not severe health complications, they should be taken into account when considering the use of *n*-3 PUFA supplementation.

### Potential Role of SPMs in Inflammation-Mediated Decline in Muscle Strength

Muscle strength may be impaired due to excessive inflammation. In athletes, inflammatory pathways together with reactive oxygen and nitrogen species are considered the most likely mechanisms responsible for overtraining syndrome in muscles, accompanied by lower muscle strength [68]. In the elderly, chronic inflammation is suggested to be main factor of sarcopenia, characterized by muscle weakness [69]. A growing body of data has indicated that EPA and DHA derivatives, SPMs, through limiting inflammation, may promote muscle strength [70,71].

Depending on the group, SPMs are characterized by different mechanisms of action, and some of them are described below. Resolvins D derived from DHA, through interaction with GPR32 receptor, inhibit the production of proinflammatory cytokines, such as TNF-α and IL6. The same cytokines are targeted for EPA-derived Resolvins E; however, they act through different receptors: E series receptor (ERV) and chemerin receptor 23 (ChemR23). The action of Protectins derived from DHA and DPA, based on regulating neutrophils and macrophages, reduction in TNF-α levels and increase in interferon-γ (IFNγ) levels. In contrast, DHA-derived Maresins, through TLR4 receptor inhibition, downregulates NF-kB and upregulates orphan receptor alpha-related RAR (RORα), resulting in the upregulation synthesis of a specific enzyme, Lipoxygenase 12 (12-LOX), and anti-inflammatory effects. Moreover, through a yet unknown mechanism, they can interact with Nrf2 and PPARs and downregulate various interleukins or affect LGR6 receptor [72].

Study conducted on rodents revealed a reduction in the degree and length of inflammation, an enhancement in the regeneration of myofiber growth, and an improvement in the recovery of muscle strength as a result of the administration of Resolvin D1 (RvD1) after myofiber injury [70]. This is in line with more recent data, where the local application of stable isomer of RvD1 after volumetric muscle loss enhanced muscle regeneration, improved muscle function, and reduced pain sensitivity [71]. The results of these studies and the potential for other SPMs to suppress inflammation suggest that they may have future applications in human studies regarding muscle strength.

## 5. Limitations of Existing Studies

There are limitations of many of the studies conducted to date, both in adults and in the elderly, that may affect the final results. These include the lack of external control (e.g., by a trainer) over the training performed by participants, the non-adherence or cessation of which can affect the outcomes [73]. The same is true for diet: for example, too low/high dietary energy intake or insufficient/too high protein supply can affect changes in muscle strength [74,75], which suggests the necessity to use a standardized diet for all participants.

The length and dose of *n*-3 PUFA supplementation appear to be another essential aspect, due to the incorporation of EPA + DHA into target tissues, which would be reflected in erythrocyte membranes and the increase in the O3I. In a review by Dempsey et al., it was summarized that, in order to raise O3I to the so-called target range (O3I ≥ 8%), at least a 12-week supplementation period of 1000–1500 mg/day of EPA and DHA would be required, while individuals with low O3I levels (O3I < 4%) would need a longer supplementation period/higher doses [76]. This is in line with a study in which a 12-week supplementation with a total dose of 3.2 g of EPA + DHA (2234 mg of EPA and 916 mg of DHA) increased O3I from 5.8% to 11.6% in amateur runners [77].

## 6. Future Directions

Future research on the application of EPA and DHA in the context of muscle strength in humans should include a rigorous methodology. This includes: training supervision and standardized diet for all participants, which will allow to exclude the influence on muscle strength factors other than EPA and DHA, as well as a sufficiently long and high dose of supplementation. The use of a muscle biopsy, which would assess the amount of *n*-3 PUFAs that will incorporate into the skeletal muscles and will potentially correlate with human physiological or functional outcomes, should also be considered in future experiments [78]. Exploring the effects of *n*-3 PUFAs on neuromuscular functions, which can possibly mediate changes in muscle strength [21,46], is also an aspect deserving further attention. Finally, the discovery of SPMs and their properties to suppress inflammation and initial reports suggesting their effectiveness in recovering muscle strength after injuries in animal models [70,71] may represent the beginning of research into the application of SPMs in inflammation-induced decline in muscle strength in humans.

## 7. Conclusions

EPA and DHA may have a small yet significant effect on preserving and/or increasing muscle strength in both healthy adults and the elderly. The paucity of research in this area including mechanisms of action generates the need for further investigations. Rigorous methodology essential to capture the small changes that are potentially induced by EPA and DHA during strength training seems to be critical factor in these studies. The promising results of using *n*-3 PUFA derivatives, SPMs, in the context of muscle strength in animal models, suggest the rationale for undertaking in future similar studies on humans.

## Figures and Tables

**Table 1:** EPA and DHA contents (mg/100 g food) in a selection foods. Based on Calder [1].

FOOD	EPA	DHA
mackerel	710	1100
salmon	500	1300
canned sardines	890	680
herring	510	690
trout	230	830
crab	470	450
cod	80	160
lamb	30	20
chicken	10	30
pork	10	10
beef	20	0

**Table 2:** The most important trials in this field.

Authors, Year of Publication	Participants	Supplementation Scheme	Exercise/Intervention	Findings
**Studies On Adults**				
Heileson et al., 2023 [21]	21 recreationally trained men and women	2275 mg EPA + 1575 mg DHA a day or placebo through 10 week of RET	1RM_SQT,_ 1RM_BP_	↑ absolute and relative 1RM_BP,_ and relative 1RM_SQT_ in O-3 group vs placebo
Philpott et al., 2019 [22]	20 resistance-trained men	2000 mg EPA + 2000 mg DHA + 18 g carbohydrate and 5 g protein a day or placebo over a 6 week, during which 2 weeks of a 60% reduced energy intake were included	1 RM leg press and leg extension, leg isokinetic/ecentric MVC	↑ 1 RM leg extension for the non-dominant leg in O-3 group vs placebo; no difference in MVC between groups
Lewis et al., 2015 [34]	30 male athletes	375 mg EPA, 230 mg DPA, 510 mg DHA a day or placebo for 21 days	quadriceps MVC force, vastus lateralis EMG	unclear effect on MVC force, ↑ vastus lateralis EMG in O-3 group vs placebo
Lewis et al., 2017 [35]	30 active men	375 mg EPA, 230 mg DPA, 510 mg DHA, and 5000 IU retinyl palmitate and vitamin D3 or placebo for 2 weeks of sprint interval training	quadriceps MVC force	no significant difference between groups
Crestani et al., 2017 [36]	15 physically active men	~1400 mg of *n*-3 PUFAs a day or placebo for 28 days	1 RM of knee extension and maximum repetition of knee extension with 70% of 1RM load pre and post an incremental running protocol	↑ 1RM of knee extension in O-3 group vs placebo
Gravina et al., 2017 [37]	13 trained men and women	100 mg/kg/day of *n*-3 PUFAs (each 1000 mg contained 70% EPA, 20% DHA, 2 mg DPA and 0.02 mg vit. E) or placebo during soccer training	1RM knee extensor strength, vertical jump power	no significant difference between groups
**Studies On the Elderly**				
Xu et al., 2022 [23]	187 older adults	1340 mg EPA + 1070 mg DHA a day or placebo for 6 months	thigh circumference, total and appendicular skeletal mass, hand grip strength, Timed Up and Go time	↑ in all parameters in O-3 group vs placebo
Rodacki et al., 2012 [24]	45 older women	400 mg EPA + 300 mg DHA a day during 90 or 150 day of strength training or 90 day of strength training alone	peak torque and rate of torque development for knee flexor and extensor plantar and dorsiflexor	↑ in all parameters after 90 and 150 days in O-3 group compared to strength training alone
Smith et al., 2011 [38]	16 older adults of both sexes	1860 mg EPA + 1500 mg DHA or a placebo for 8 weeks	muscle protein synthesis rate, mTOR^Ser2448^ and p70s6k^Thr389^ phosphorylation in basal, postabsorptive conditions and during a hyperaminoacidemic–hyperinsulinemic clamp	↑ in all parameters in O-3 group vs placebo during a hyperaminoacidemic–hyperinsulinemic clamp
Jang et al., 2020 [39]	125 adults of both sexes	correlation of serum *n*-3 PUFAs with sarcopenia indicators (muscle mass, hand grip strength, repeated chair stands, standing balance, and walking speed)	-	↑ serum *n*-3 PUFAs level was significantly associated with a greater grip strength
Krzymińska-Siemaszko et al., 2015 [40]	53 older adults of both sexes	660 mg EPA + 440 mg DHA + 200 mg other *n*-3 PUFAs + 10 mg of vit. E or placebo for a 12 weeks	hand grip strength, Time Up and Go test	no significant differences between groups
Hab et al., 2022 [41]	61 older adults of both sexes	2190 mg EPA + DHA in algal oil + 27 g protein as whey protein and high-protein diet (1.2–1.5 g/kg) or placebo combined with vibration and home- based resistance exercise	muscle power (watt/m^2^) during chair rise test chair rise test (time), knee extension strength grip strength	↑ muscle power during chair rise test (only in men)

EPA = eicosapentaenoic acid; DHA = docosahexanoic acid; RET = resistance exercise training; 1RM = one maximum repetition; SQT = squat; BP = bench press; MVC = maximum voluntary contraction; PUFA = polyunsaturated fatty acids; O-3 = omega-3; ↑ = statistically significant increase.
